# Exploration of KCNJ5 Somatic Mutation and CYP11B1/CYP11B2 Staining in Multiple Nodules in Primary Aldosteronism

**DOI:** 10.3389/fmed.2022.823065

**Published:** 2022-04-12

**Authors:** Jing Xie, Cui Zhang, Xuefeng Wang, Yiran Jiang, Luming Wu, Lei Ye, Xuan Wang, Wen Xie, Haimin Xu, Weiqing Wang

**Affiliations:** ^1^Department of Pathology, Shanghai Jiao Tong University School of Medicine, Shanghai, China; ^2^Department of Pathology, Ruijin Hospital, Shanghai Jiao Tong University School of Medicine, Shanghai, China; ^3^Department of Endocrine and Metabolic Diseases, Ruijin Hospital, Shanghai Institute of Endocrine and Metabolic Diseases, Shanghai Jiao Tong University School of Medicine, Shanghai, China; ^4^Department of Clinical Laboratory, Ruijin Hospital, Shanghai Jiao Tong University School of Medicine, Shanghai, China; ^5^Department of Pathology, Shanghai Pulmonary Hospital, Tongji University School of Medicine, Shanghai, China; ^6^Department of Pathology, Zhongnan Hospital of Wuhan University, Wuhan, China

**Keywords:** KCNJ5, CYP11B2, CYP11B1, primary aldosteronism, unilateral multiple nodules

## Abstract

**Objective:**

Unilateral primary aldosteronism (PA) includes aldosterone-producing adenoma (APA), unilateral adrenal hyperplasia, and unilateral multiple nodules. The correlation of multiple nodules, especially genotypic and pathological characteristics, remains unknown. KCNJ5 mutation accounts for 60–80% of unilateral PA, so we aimed to explore the correlation of KCNJ5 somatic mutation and CYP11B1/CYP11B2 staining in multiple nodules in unilateral PA.

**Design and Methods:**

A total of 56 microdissected nodules from 24 patients with unilateral PA were included. We assessed somatic KCNJ5 mutations, immunohistochemistry for aldosterone synthase (CYP11B2)/cortisol synthase (CYP11B1), and histological cellular composition of nodules together with adjacent adrenal cortical statements.

**Results:**

KCNJ5 mutations were identified in 17 (17/56, 30.4%) nodules from 11 adrenals (11/24, 45.8%). All KCNJ5-mutant nodules were positive for CYP11B2 staining, 6 cases (6/11) had only one KCNJ5-mutant nodular, and the other 5 cases (5/11) had more than one KCNJ5-mutant nodules. Three cases (3/11) had different KCNJ5 mutations in individual nodules. Compared with KCNJ5-positive adrenals, the cortices adjacent to the nodules in KCNJ5-negative adrenals showed significant proliferation (*p* = 0.004). CYP11B2/CYP11B1 expression patterns revealed great heterogeneity in intensity and range both in KCNJ5-mutant nodules and KCNJ5-WT ones.

**Conclusion:**

There is great heterogeneity among nodules from patients with unilateral PA. Countable nodules could be considered as multiple APAs, featuring somatic KCNJ5 mutation, positive CYP11B2 staining, and lack of adjacent cortical proliferation in unilateral multiple nodules.

## Introduction

Primary aldosteronism (PA) is characterized by hypertension, hypokalemia, increased plasma aldosterone, and suppressed renin levels and is the most potentially curable form of secondary hypertension, occurs in 6–10% of hypertensive patients (1–3). PA is divided into the unilateral and bilateral diseases by adrenal vein sampling (AVS). Unilateral PA is best treated by adrenalectomy, whereas bilateral PA requires treatment with mineralocorticoid receptor antagonists (4). In past years, most unilateral PAs are aldosterone-producing adenomas (APAs), and unilateral adrenal hyperplasia (UAH) is considered to be rare. However, an increasing number of studies have focused on UAH; in our center, we previously reported that UAH accounted for 19% of PA cases (5). Immunohistochemical analysis of CYP11B1 (cortisol synthase) and CYP11B2 (aldosterone synthase) provides important functional information and can aid in the histopathological diagnosis of unilateral nodules (6–9). Especially, an international group of pathologists and adrenal experts has published the histopathology of PA and reached a consensus in standardizing the histopathologic features of adrenals from patients with PA (10), UAH on histopathology is divided into multiple adrenocortical micronodules (MN) and diffuse hyperplasia of zona glomerulosa (DH) (11). However, many nodules in MN do not exhibit aldosterone-producing zona glomerulosa (ZG)-like cells (small, with a high nuclear–cytoplasmic ratio and a smaller lipid content), as would be expected, but zona fasciculata (ZF)-like cells (large clear cells with lipid-laden cytoplasm and small nuclei) normally produce cortisol (12–14). Small extra nodular cell clusters are observed with strong CYP11B2 expression and no CYP11B1 expression in the surrounding cortical tissue (7). Therefore, the pathogenesis of unilateral MN remains unknown.

In addition to histopathology and immunohistochemistry, molecular analysis is also important for pathogenesis. KCNJ5, first described in a case of familial hypertension III by Choi et al. (15), induces the activation of aldosterone synthesis and has been implicated in promoting the growth of aldosterone-secreting cells into APAs (8). Other mutations include CACNA1D, ATP1A1, and ATP2B3 (15, 16). In China, KCNJ5 mutations occur in up to 70.7% of APAs (17) and account for most unilateral adrenal diseases. APAs appear as a single well-circumscribed nodule with various degrees of adrenal cortex remodeling, i.e., atrophic, with a diffuse hyperplasia or nodular hyperplasia (18, 19).

Heterogeneity in PA has long been a focus of research and is a lasting topic of interest in this field. It was reported that different somatic gene mutations occurred at different CYP11B2-positive areas within the same tumor (20). There were few studies that focused on KCNJ5 mutation in the unilateral multinodular lesions. Especially whether KCNJ5 mutation occurred in different nodules within one adrenal gland remains unclear. In this study, we assessed the phenotypic and genotypic characteristics of 24 patients with unilateral multiple nodules in this topic.

## Materials and Methods

### Subjects

We enrolled 24 patients with unilateral PA who underwent unilateral adrenalectomy and were diagnosed as multiple nodules by hematoxylin and eosin (HE) staining in Ruijin Hospital affiliated with Shanghai Jiao Tong University School of Medicine from 2016 to 2019. Informed consent was obtained from all participants in this study. Patients were diagnosed with PA by saline infusion test according to Endocrine Society Clinical Practice Guidelines 2016 (4). All patients underwent adrenal computed tomography (CT) scans. A unilateral CT performance was defined as a unilateral adenoma (>10 mm), with a smooth appearance observed for the contralateral glands. Bilateral CT was used to detect bilateral adrenal hyperplasia or nodules on CT. Adrenal venous sampling (AVS) was performed by an experienced radiologist. Cannulation was considered successful if the cortisol adrenal vein:cortisol peripheral vein ratio was greater than 3 without adrenal corticotropic hormone (ACTH) stimulation (17). Twenty-one patients underwent AVS with unilateral lateralization, and 3 patients were diagnosed with unilateral PA by CT scan. All the adrenals from these patients were identified to multiple nodules under microscopy.

### Pathological Analysis

Histological examination of the removed adrenals was performed by two experienced pathologists. All adrenal gland examinations included gross observation and microscopic assessment.

Specimen were well prepared for all adrenal glands, embedded in paraffin, cut into 3 μm thick slices, stained with H&E, and then were analyzed to obtain a diagnosis of the multinodular lesion. The cellular composition was determined by examining known features: ZF, i.e., large and lipid-laden clear cells; ZG, i.e., small and clear cells; and zona reticularis (ZR), i.e., large and acidophilic lipid-deleted compact cells or in combination. The details of the adjacent cortex were determined as follows: atrophy or normal (0), slight or focal hyperplasia (1+), medium hyperplasia (2+), and remarkable/diffuse hyperplasia (3+) (Supplementary Figure 1). Aldosterone-producing cell clusters (APCCs) were defined as “CYP11B2-positive lesion (<10 mm diameter) composed of ZG cells located beneath adrenal capsule that do not differ in morphology from adjacent adrenocortical cells by H&E staining” according to the consensus in 2020 (10).

### Immunohistochemical Analysis

We performed immunohistochemistry of CYP11B1 and CYP11B2 in all the nodules selected by microscopy to assess the cortisol- and aldosterone-secreting function of the adrenals, respectively. Immunohistochemistry was performed according to previously described protocols (7). The antibodies for CYP11B1 (monoclonal mouse anti-human CYP11B1, clone H-11, 1:100, SANTA CRUZ) and CYP11B2 (monoclonal mouse anti-human CYP11B2, clone 41-17B, 1:100, EMD Millipore) were used for immunohistochemistry on formalin-fixed paraffin-embedded sections using Chem Mate ENVISION kits (Dako).

Semiquantitative immunoreactivity scoring was used to investigate the functional significance of CYP11B1 and CYP11B2, which was assessed by the McCarty H-score (ranging from 0 to 300) with all tumors examined under a 20 × objective. In each field, the percentage of immunopositive cells was assessed and then multiplied by a factor from 0 to 3 according to the intensity of immunopositivity (21). The relative immunointensity of specific immunoreactivity was characterized as not present (0), weak but detectable above the control (1+), distinct (2+), or very strong (3+) (Supplementary Figures 2, 3).

### Genotyping

All visible nodules were demarcated on the H&E-stained slides by a pathologist using a marker pen. Six 10-μm sections were manually microdissected for each nodule. Genomic DNA was prepared from all separate nodules and normal adrenals by overnight digestion with proteinase K and analyzed separately. KCNJ5 somatic mutations were performed by Sanger sequencing. DNA fragments were sequenced for PCR amplification using previously reported primers: 5’-TTGGCGACCAAGAGTGGATTCCTT-3’ and 5’-CACCATGAAGGCATTGACGATGGA-3.

### Statistical Analysis

Statistical Analysis System (SAS) 9.4 was used for the statistical analyses. Continuous variables are presented as the means ± standard deviation (SD) if they were normally distributed and P25 – P75 if they were not normally distributed. Categorical variables are presented as numbers (%). Continuous and categorical variables were compared using Student’s *t*-test and Fisher’s exact test, respectively. Statistical significance was defined as an α-level less than 0.05 on two-sided tests.

## Results

The 24 adrenal glands that were analyzed contained 56 nodules in total (2–4 nodules for each gland), which were all examined separately. There were no significant differences in sex, age, blood pressure (BP), and potassium level between patients with KCNJ5 mutations and those without any KCNJ5 mutations. However, the age in the KCNJ5 mutation group was younger than that of the KCNJ5-wild type (KCNJ5-WT) group. Serum cortisol, plasma aldosterone, and renin activity were similar between the two groups; however, plasma ACTH was lower in KCNJ5-mutant patients (Table 1).

**TABLE 1:** Clinical features of 24 patients with primary aldosteronism (PA).

Variables	KCNJ5-wild type (*n* = 13)	KCNJ5-mutant (*n* = 11)	*P*
Age (years)	52.4 ± 12.0	43.7 ± 11.8	0.09
Female (*n*, %)	4 (30.8)	5 (45.5)	0.46
Systolic BP (mmHg)	166.5 ± 19.7	169.0 ± 32.2	0.82
Diastolic BP (mmHg)	97.3 ± 9.3	100.7 ± 14.9	0.50
Serum potassium (mmol/L)	2.60 ± 0.70	2.77 ± 0.58	0.53
Aldosterone (ng/dL)	537.2 ± 350.8	462.5 ± 185.6	0.54
PRA (ng/mL/h)	0.40 ± 0.23	0.23 ± 0.23	0.15
Serum cortisol (8 a.m.)	11.5 ± 2.7	10.3 ± 3.0	0.32
ACTH (pg/mL)	38.5 ± 9.9	29.1 ± 9.7	0.04

*Values are arithmetic (± SD) or the number of subjects (%).*

All KCNJ5 mutations were present in CYP11B2-positive cells in 11 cases (11/24, 45.8%) comprising 17 nodules (17/56, 30.4%). The average size of KCNJ5-mutant nodules was 1.12 cm, which was larger than that of KCNJ5-WT (0.83 cm). However, no significant differences in the cell components were found between nodules in the KCNJ5-mutation and KCNJ5-WT groups, the KCNJ5 mutations were occurred in ZF-dominant nodules (9/17 in ZF-cells, 7/17 in ZF + ZR cells). The KCNJ5-WT nodules exhibited adjacent cortex hyperplasia (32/39) when compared to the KCNJ5-mutant nodules (7/17) (*p* = 0.004). Interestingly, the decrease in CYP11B1 expression was more remarkable than the increase in CYP11B2 (*p* = 0.009; Table 2).

**TABLE 2:** Characteristics of 56 nodules with and without KCNJ5 mutations in 24 patients with primary aldosteronism (PA).

Characteristic	KCNJ5-Wild type (*n* = 39)	KCNJ5 mutant (*n* = 17)	*P*
Size (cm)	0.83 ± 0.51	1.12 ± 0.61	0.07
CYP11B1	123.0 ± 82.7	47.5 ± 44.3	0.009
CYP11B2	225.1 ± 108.8	258.5 ± 41.0	0.18
Cell components of nodules			
ZG + ZR	0 (0)	1 (5.9)	0.30
ZG + ZF	1 (2.6)	0 (0)	0.70
ZF	16 (41.0)	9 (52.9)	0.56
ZF + ZR	18 (46.2)	7 (41.2)	0.78
ZR + ZF	4 (10.3)	0 (0)	0.30
Hyperplasia in adjacent cortex	32 (82.1)	7 (41.2)	0.004

*Values are arithmetic (± SD) or the number of subjects (%).*

In 5 cases (cases 5, 8, 11, 19, and 24), multiple KCNJ5 mutations were found in each adrenal nodular, 2 cases (cases 11 and 24) had the same mutations in different nodules within one gland, and 3 cases (cases 5, 8, and 19) had different mutations in separate nodules (Table 3). The hotspot mutations of KCNJ5 included 503 (168) T > G, 451 (151) G > A, and 451 (151) G > C. The nodules in one gland carrying the same mutations showed varied expressions of CYP11B2 but negative expressions of CYP11B1. While the nodules harbored different mutations in cases 5, 8, and 19, the situation is seemed different. In these cases, KCNJ5-mutated nodules showed uniform expression of CYP11B2 but a varied expression of CYP11B1.

**TABLE 3:** Characteristics of 56 nodules.

Case	Size (cm)	Spots of KCNJ5 mutation	Expression of B1	Expression of B2	Cell components of nodules	Status of adjacent cortex
1	N1 0.6	Neg	0	100% 3 +	ZG + ZF	2
	N2 0.5	Neg	70% 2+	2% +	ZF + ZR	2
2	N1 1.2	451 (151)G > A	50% 1 + 40% 2 +	60% 2 +	ZF	0
	N2 0.8	Neg	80% 2 +	20% 2 +	ZF + ZR	0
3	N1 1.0	Neg	5% 2 +	100% 3 +	ZF + ZR	2
	N2 0.6	Neg	80% 3 +	20% 1 +	ZF + ZR	2
4	N1 0.4	Neg	50% 2 +	0	ZR + ZF	2
	N2 0.5	Neg	50% 2 +	5% 2 +	ZF + ZR	2
	N3 0.6	Neg	80% 3 +	0	ZF + ZR	2
	N4 0.3	503 (168) T > G	100% 1 +	90% 3 +	ZF + ZR	2
5	N1 0.6	503 (168)T > G	30% 1 +	100% 3 +	ZF + ZR	0
	N2 1.5	451 (151)G > A	30% 1 +	100% 3 +	ZG + ZF	0
6	N1 0.8	Neg	0	0	ZF	1
	N2 1.7	Neg	20% 1 +	0	ZF	1
	N3 0.5	Neg	40% 2 +	0	ZF	1
	N4 0.8	Neg	40% 2 +	60% 3 +	ZF + ZR	1
7	N1 2.2	Neg	20% 1 +	80% 3 +	ZF	0
	N2 0.8	451 (151)G > A	0	80% 3 + 20% 2 +	ZF	0
8	N1 0.7	451 (151)G > A	10% 1 +	70% 3 +	ZF	0
	N2 1.5	503 (168)T > G	70% 1 +	80% 3 + 20% 2 +	ZF + ZR	0
9	N1 0.8	Neg	100% 1 +	0	ZF	2
	N2 0.6	Neg	100% 1 +	0	ZF + ZR	2
	N3 1.7	Neg	80% 1 +	100% 3 +	ZF	2
10	N1 0.5	503 (168)T > G	5% 2 +	100% 3 +	ZF	2
	N2 0.3	Neg	10% 3 +	100% 3 +	ZF	2
11	N1 2.0	503 (168)T > G	0	80% 3 + 10% 2 +	ZF + ZR	0
	N2 1.5	503 (168)T > G	0	90% 3 + 10% 2 +	ZF + ZR	0
	N3 0.6	503 (168)T > G	0	50% 3 + 50% 2 +	ZF + ZR	0
12	N1 1.0	451 (151)G > A	10% 1 +	90% 3 + 10% 2 +	ZF	0
	N2 0.6	Neg	60% 3 + 20% 2 +	0	ZF	0
13	N1 0.3	Neg	100% 1 +	0	ZR + ZF	0
	N2 0.4	Neg	100% 1 +	0	ZR + ZF	0
14	N1 2.0	503 (168)T > G	0	90% 3 + 10% 2 +	ZF + ZR	1
	N2 0.8	Neg	100% 3 +	0	ZF + ZR	1
15	N1 0.6	Neg	0	100% 3 +	ZF + ZR	2
	N2 0.4	Neg	20% 1 +	0	ZF + ZR	2
	N3 0.4	Neg	50% 3 + 40% 2 +	0	ZF + ZR	2
16	N1 1.5	Neg	60% 2 +	80% 3 + 10% 2 +	ZF + ZR	1
	N2 1.0	Neg	70% 1 + 10% 2 +	60% 3 + 20% 2 +	ZF + ZR	1
17	N1 0.8	Neg	80% 3 +	70% 1 +	ZR + ZR	3
	N2 0.7	Neg	70% 2 + 30% 1 +	30% 3 +	ZF + ZR	3
18	N1 1.0	Neg	10% 3 + 90% 2 +	10% 3 + (on the periphery)	ZF	2
	N2 1.5	Neg	10% 1 +	90% 3 + 10% 2 +	ZF + ZR	2
19	N1 0.8	Neg	30% 2 +	90% 3 + 10% 2 +	ZF	3
	N2 0.7	451 (151)G > A	30% 1 +	80% 3 + 10% 1 +	ZF	3
	N3 0.4	451 (151)G > C	30% 1 +	80% 3 + 10% 1 +	ZF	3
20	N1 1.0	Neg	0	100% 3 +	ZF + ZR	2
	N2 0.7	Neg	20% 1 +	0	ZF + ZR	2
21	N1 0.5	Neg	0	100% 3 +	ZF	3
	APCC (0.2)	Neg	0	100% 3 +	ZF	3
22	N1 0.5	Neg	0	80% 3 +	ZF	0
	N2 2.5	Neg	0	70% 3 +	ZF	0
23	N1 1.0	Neg	100% 1 +	100% 3 +	ZF	3
	N2 0.8	Neg	100% 1 +	100% 3 +	ZF	3
24	N1 2.2	503 (168)T > G	0	50% 3 + 40% 2 +	ZF	1
	N2 1.5	503 (168)T > G	0	70% 2 + 10% 3 +	ZF	1

In 5 cases (2, 7, 10, 12, and 14), every gland contained 2 nodules and only one of them had a KCNJ5 mutation (Table 3). The mutation site involved 503 (168) T > G and 451 (151) G > A. Compared to KCNJ5-WT nodules, KCNJ5-mutant nodules were prone to be larger ones (4/5 cases, cases 2, 10, 12, and 14). KCNJ5 mutations unlikely occurred in nodules that were CYP11B1-positive (intense > 2+ and range > 50%), even if CYP11B2 was partly positive (intense ≥ 2+, any range) (2/3 cases, cases 2, 12, and 14). Moreover, KCNJ5 mutations occurred in CYP11B1-negative nodules, nevertheless, the expression of CYP11B2 varied (cases 10 and 14).

**FIGURE 1:**
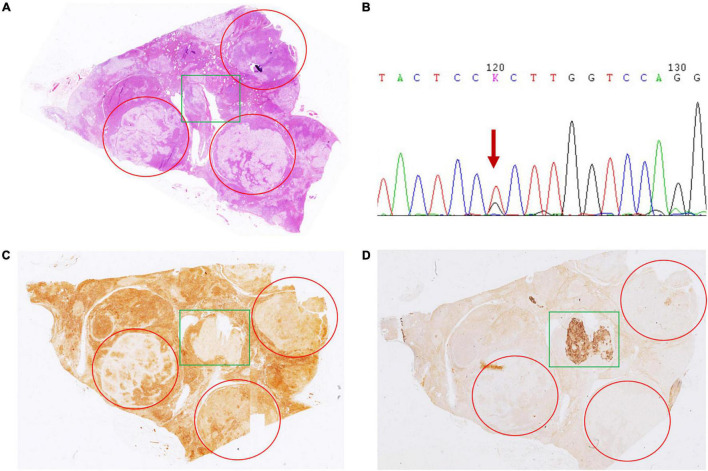
Hematoxylin and eosin (H&E) and immunohistochemical staining of CYP11B1 and CYP11B2 in case 4. **(A)** Innumerable nodules with intensive hyperplasia of the gland with 3 well-demarcated nodules by 0.4, 0.5, and 0.6 cm in diameter independently (marked by red circles). Irregular zone with CYP11B1 **(C)** weak staining and CYP11B2 **(D)** strong expression (marked by green rectangles). Irregular zone with CYP11B2 strong expression harbored KCNJ5 mutation of 503 (168) T > G **(B)**.

In case 4, gross examination showed intensive hyperplasia of the gland with 3 well-demarcated nodules that were 0.4, 0.5, and 0.6 cm in diameter independently. Microscopically, countless micronodules were observed, and three nodules could be defined correspondingly (Figure 1A). Immunohistochemical staining indicated moderate to strong CYP11B1 expressions and negative CYP11B2 expressions (Figures 1C,D). Surprisingly, an irregular zone of 3 mm × 3 mm with weak CYP11B1 and strong CYP11B2 expressions was found (Figures 1C,D). This APCC-like pattern carried the KCNJ5 mutation of 503 (168) T > G (Figure 1B).

In the remaining cases, no KCNJ5 mutation was detected. CYP11B2/CYP11B1 expression patterns also revealed great heterogeneity in intensity and range (Table 3). CYP11B2 was strongly expressed in double-nodular glands in 4 cases (16, 21, 22, and 23). Among them, CYP11B1 expression varied in case 16, while in the other 3 cases (21, 22, and 23), CYP11B1 staining was almost negative. The 5 cases (1, 3, 9, 15, and 20) possessed one CYP11B2-positive nodule with completely negative or sporadically CYP11B2-positive signals in the other nodules, CYP11B1 expression varied in these CYP11B2-negative nodules.

**FIGURE 2:**
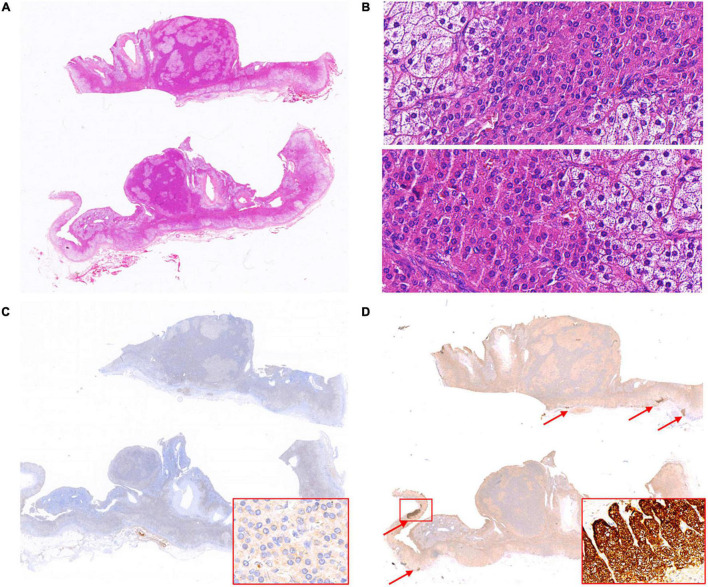
Hematoxylin and eosin (H&E) and immunohistochemical staining of CYP11B1 and CYP11B2 in case 13. Two well-demarcated nodules in adrenal (**A**, up and down); two nodules both composed of acidophilic cells and clear cells (**B**, 400× magnification for nodules respectively, up and down). Weak expression of CYP11B1 (**C**, 400× magnification for insert), completely negative staining of CYP11B2 in both nodules, several APCCs detected in the adrenal cortex (**D**, indicated by red arrows, 200× magnification for insert).

In case 13, two well-demarcated nodules that are composed of acidophilic cells and clear cells (Figures 2A,B) showed completely negative staining for CYP11B2 and weak expression of CYP11B1 (Figures 2C,D). Several APCCs beneath the adrenal capsule were detected (Figure 2D). Though these CYP11B2-positive/CYP11B1-negative cell clusters might be markedly exhibited by CYP11B2 staining, they could hardly be detected by H&E staining.

**FIGURE 3:**
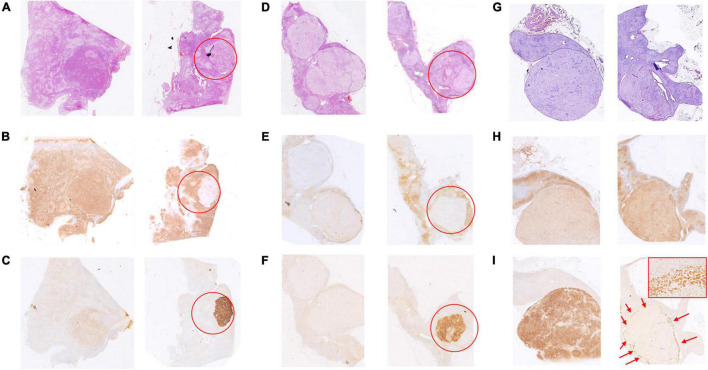
Hematoxylin and eosin (H&E) and immunohistochemical staining of CYP11B1 and CYP11B2 in cases 17, 6, and 18. In case 17, one obscure nodular mainly with acidophilic cells (**A**, left), the other one with a mixture of acidophilic cells and clear cells (**A**, right, marked by the red circle). One-third of the nodules are positive for CYP11B2 (**C**, marked by the red circle) and weak positive for CYP11B1, moderate expression for CYP11B1 in the rest zone of the nodule (**B**, marked by red circle). In case 6, one of 4 clear cell nodules (0.8 cm in diameter) (**D**, marked by the red circle), focal CYP11B2-positive and CYP11B1-negative area by 4 mm × 3 mm **(E,F)**, moderate expression of CYP11B1 in the rest of nodule **(E)**. In case 18, 2 clear cell-dominant nodules (**G**, left and right), the rare annular-pattern of CYP11B2 expression on the periphery of the smaller nodule (**I**, indicated by red arrows, 200 × magnification for insert), moderate expression of CYP11B1 (**H**, right), strong expression of CYP11B2 (**I**, left), and slight expression of CYP11B1 (**H**, left) in the bigger one.

There were 3 rare CYP11B2/CYP11B1 expression patterns that deserve further description. In case 17, the adrenal had 2 nodules with a mixture of acidophilic cells and clear cells by 0.7 and 0.8 cm in diameter, respectively (Figure 3A). The CYP11B2-positive/CYP11B1-weak positive area was occupied by approximately one-third of the clear cell dominant nodule; the rest of the nodule was CYP11B2-negative/CYP11B1-moderate positive (Figures 3B,C). A similar situation was observed in case 6 (Figure 3D); CYP11B2-positive expression was focused on an area of 4 mm × 3 mm in size on nodule 4 (0.8 cm), and this nodular displayed moderate CYP11B1 expression in the “CYP11B2-negative” zone (Figures 3E,F). The situation was more interesting in case 18. In addition to the nodule 2 of CYP11B2-positive, CYP11B2 staining was only demonstrated in the annular area on the periphery of nodule 1, and the expression of CYP11B1 was moderate (Figures 3G–I).

## Discussion

Unilateral multiple nodule is considered a rare subtypes of PA that is clinically characterized by unilateral adrenal hypersecretion of aldosterone and morphologically characterized by multiple adrenal nodules (22). The pathogenesis and correlation of different nodules were interesting and meaningful. In this study, we describe additional characteristics of multinodular adrenals that include KCNJ5 mutation and CYP11B1/CYP11B2 expression. Based on the microscopic morphological manifestations, immunohistochemistry, and molecular characteristics, some of the multiple nodules are consistent with a diagnosis of “adenoma.” Therefore, we proposed “double adenomas” or “multiadenomas” as follows: multiple well-demarcated nodules; lack of adjacent cortical proliferation; KCNJ5 mutant; positive expression of CYP11B2; and negative/weak expression of CYP11B1. Our research has proved that multiple nodules in one adrenal could harbor different KCNJ5 mutation sites. This finding demonstrated that the nodules were originated from different clones and further suggested the possibility of multiple adenomas. The expressions of CYP11B2 were different among multiple KCNJ5-mutant nodules. This finding indicated that different nodules might contribute jointly to abnormal aldosterone synthesis and secretion. In addition, it has been reported that aldosterone adenoma occurred simultaneously (or successively) in the same or opposite adrenal glands (23, 24). Therefore, such multinodular lesions are obviously different from traditional “nodular hyperplasia,” and they should be distinguished from the subtype of multiple nodules (formally known as nodular hyperplasia).

KCNJ5 mutations were present in 35–60% of the glands (50% in APAs), and ATP2B3, ATP1A1, and CACNA1D mutations were found in cases of both solitary adenoma and nodular hyperplasia (20, 25–28). In our study, KCNJ5 mutations were found to exist in 45.8% of our 24 unilateral adrenalectomy samples, which is lower than our previous report in unilateral PA (17). All KCNJ5 mutations occurred in CYP11B2-positive nodules mostly composed of ZF-like cells, which was consistent with previous reports (25, 29). The KCNJ5 mutations in our study were found more often in younger patients without sex differences. KCNJ5 mutations were mostly detected in the larger nodular of unilateral multinodular adrenal specimens in five cases. This result is consistent with previous reports (30). In addition, our results showed that nodules without surrounding adrenocortical hyperplasia or with mild hyperplasia were more likely to have KCNJ5 mutations than nodules with obvious hyperplasia, which could be observed by pathological examination or CT scanning. This result suggests that we may need to carefully evaluate the adrenal nodules and the adjacent cortex preoperatively to predict the KCNJ5 mutation status in patients with PA.

Compared to KCNJ5-WT nodules, we found that the expression of CYP11B2 was increased and the expression of CYP11B1 was decreased in KCNJ5-mutant nodules. This indicates that the KCNJ5 mutation increases the expression of CYP11B2, while the expression of CYP11B1 is significantly suppressed. We also found the level of ACTH was lower in KCNJ5-mutant patients, which may need further study on melanocortin 2 receptor (MC2R) in KCNJ5-mutated nodules. Therefore, abnormal aldosterone synthesis and secretion should be caused by not only the expression of CYP11B2 but also the imbalanced expression of CYP11B1 and CYP11B2, the mechanism of which needs to be further clarified.

According to the expression of CYP11B2, it is convenient for pathologists to distinguish the APA/nodule in a resected specimen of PA. However, what about the rest of the nodules with CYP11B2-negative/CYP11B1-positive, even those double negative CYP11B2/CYP11B1. More research studies are needed to determine whether CYP11B2-negative nodules are hyperplastic nodules, non-functional adenomas, or potential/silent aldosteronoma inhibited by other CYP11B2-positive nodules.

The diversity and complexity of CYP11B2 expression attract many researchers. The heterogeneous expression of CYP11B2 has been described in some studies, and some have shown that KCNJ5 mutations are only present in CYP11B2-positive cells, although the nodules consist of CYP11B2-positive cells and CYP11B2-negative cells (6, 20, 25). In our study, in nodular 2 of case 18, CYP11B2 expression was demonstrated in a rare circular pattern on the periphery of the nodule, in nodule 4 of case 6 and nodule 1 of case 17, CYP11B2 showed a partial positive area. Though these nodules are negative for KCNJ5 mutation, further investigation should be continued.

Aldosterone-producing cell clusters are described as cell nests with defined boundaries isolated from neighboring areas exhibiting remarkable CYP11B2 expression (31). In case 13, two nodules with clear borders that were similar to adenomas neither expressed CYP11B2 nor harbored KCNJ5 mutations. Several APCCs were detected in the neighboring adrenal cortex. The mechanism of this type of PA remains unclear. Some researchers assume that the cause of aldosterone hyperplasia is attributed to the multiple APCCs around CYP11B2-negative nodules. However, multiple APCCs can be found in PA and Cushing syndrome, even in normal adrenals. However, a recent report declared that KCNJ5 mutations could be detected in APCCs (32). Some researchers ascribe the negative findings to the pathologist, who failed to obtain the lesion during the specimen collection process, especially for some micronodules. In our study, all the adrenals were prepared for observation, so overlooking micronodules cannot fully explain this phenomenon. Therefore, there are likely some unknown mechanisms. Interestingly, in case 4, KCNJ5 mutation was detected in the irregular APCC-like zone instead of the 3 well-demarcated nodules. Whether those APCC-like cells are precursors to aldosterone adenoma remains to be further investigated.

There were some limitations in this study. We did not screen other mutations in the KCNJ5-WT nodules, such as ATP2B3, ATP1A1, and CACNA1D. We focus on KCNJ5 mutant and WT nodules because KCNJ5 mutation is the most common one. We can easily conclude the pathogenesis feathers of PA and we propose KCNJ5 is an important factor to make pathological diagnosis in PA. Various levels of CPY11B1 and CPY11B2 expressions illustrate the diversity and complexity in PA, the pathological diagnosis of PA is still full of challenges. In the future, we will study others mutations to improve pathological diagnoses. In conclusion, the term “multiple nodules” in PA should be reconsidered. Some nodules contained KCNJ5 mutations, positive CYP11B2 staining, and lack of adjacent cortical proliferation, which suggested the possibility of “double or multiple aldosteronomas” in PA.

## Data Availability Statement

The datasets presented in this study can be found in online repositories. The names of the repository/repositories and accession number(s) can be found in the article/Supplementary Material.

## Ethics Statement

The studies involving human participants were reviewed and approved by the Ruijin Hospital. The patients/participants provided their written informed consent to participate in this study.

## Author Contributions

JX: full access to all of the data in the study and takes responsibility for the integrity of the data and the accuracy of the data analysis. JX, CZ, and WW: concept and design, drafting of the manuscript, and statistical analysis. JX, CZ, YJ, LW, and LY: data collection. JX, CZ, XFW, XuW, WX, and HX: analysis and interpretation of data. All authors contributed to the article and approved the submitted version.

## Conflict of Interest

The authors declare that the research was conducted in the absence of any commercial or financial relationships that could be construed as a potential conflict of interest.

## Publisher’s Note

All claims expressed in this article are solely those of the authors and do not necessarily represent those of their affiliated organizations, or those of the publisher, the editors and the reviewers. Any product that may be evaluated in this article, or claim that may be made by its manufacturer, is not guaranteed or endorsed by the publisher.

## Supplementary Material

Supplementary Figure 1 Microscopical assessment of adjacent cortex. **(A)** Atrophy or normal (0); **(B)** slight or focal hyperplasia (1+); **(C)** medium hyperplasia (2+); and **(D)** remarkable/diffuse hyperplasia (3+).

Supplementary Figure 2 The assessment of CPY11B1 expression by immunohistochemistry. **(A)** Not present (0); **(B)** weak but detectable above control (1+); **(C)** distinct (2+); and **(D)** very strong (3+).

Supplementary Figure 3 The assessment of CPY11B2 expression by immunohistochemistry. **(A)** Not present (0); **(B)** weak but detectable above control (1+); **(C)** distinct (2+); and **(D)** very strong (3+).
