# Supplementary figures and images for: Exploration of KCNJ5 Somatic Mutation and CYP11B1/CYP11B2 Staining in Multiple Nodules in Primary Aldosteronism
Source: Front Med (Lausanne). 2022 Apr 12;9:823065. doi: 10.3389/fmed.2022.823065 (PMC9039053; doi:10.3389/fmed.2022.823065)

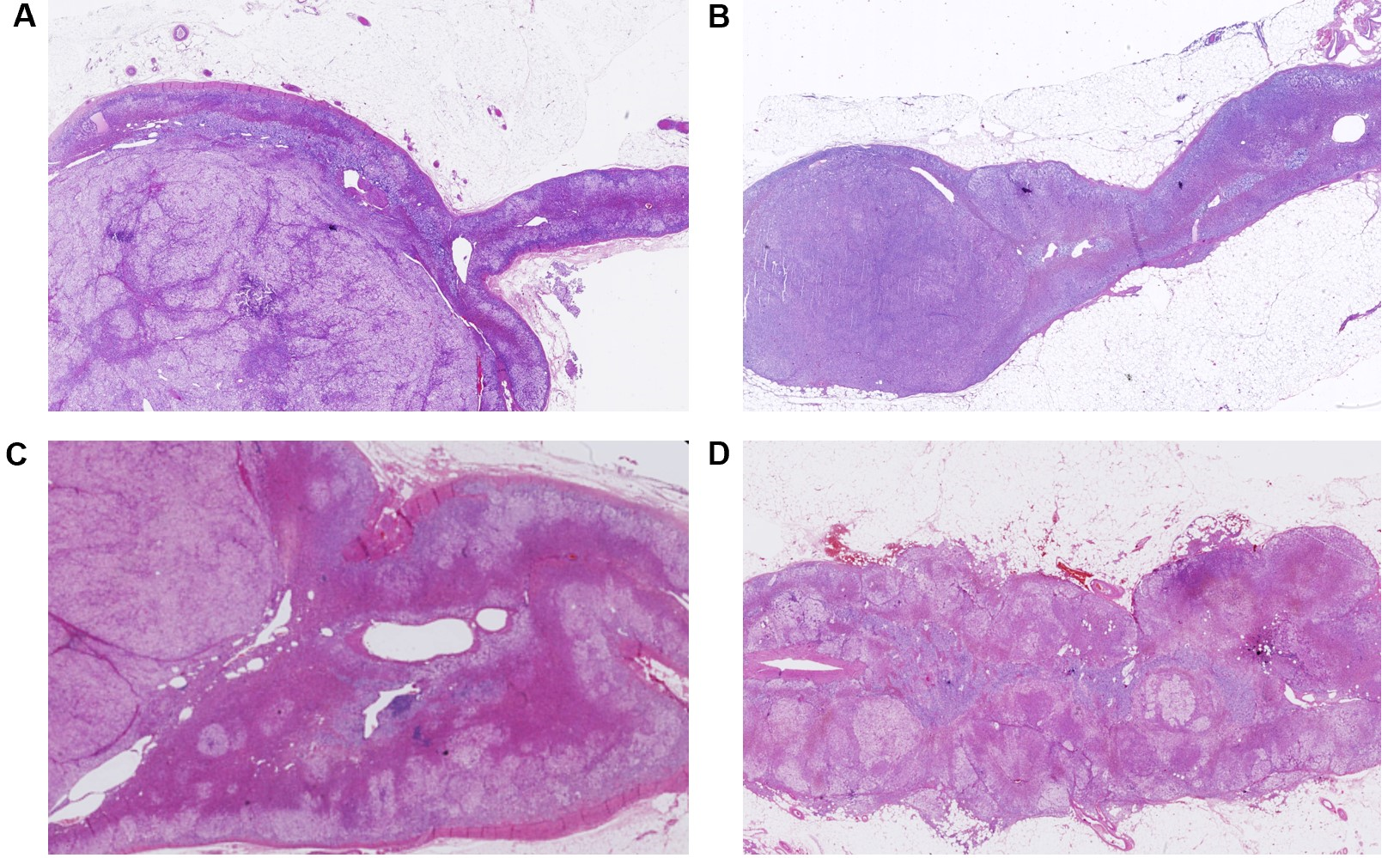

Supplement: Supplementary Figure 1 — Microscopical assessment of adjacent cortex. (A) Atrophy or normal (0); (B) slight or focal hyperplasia (1+); (C) medium hyperplasia (2+); and (D) remarkable/diffuse hyperplasia (3+). [file Image_1.tif]

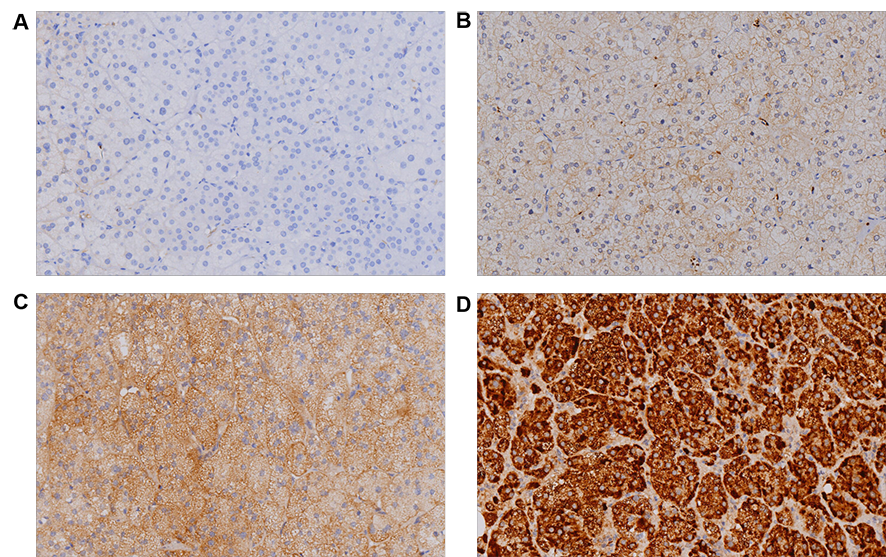

Supplement: Supplementary Figure 2 — The assessment of CPY11B1 expression by immunohistochemistry. (A) Not present (0); (B) weak but detectable above control (1+); (C) distinct (2+); and (D) very strong (3+). [file Image_2.tif]

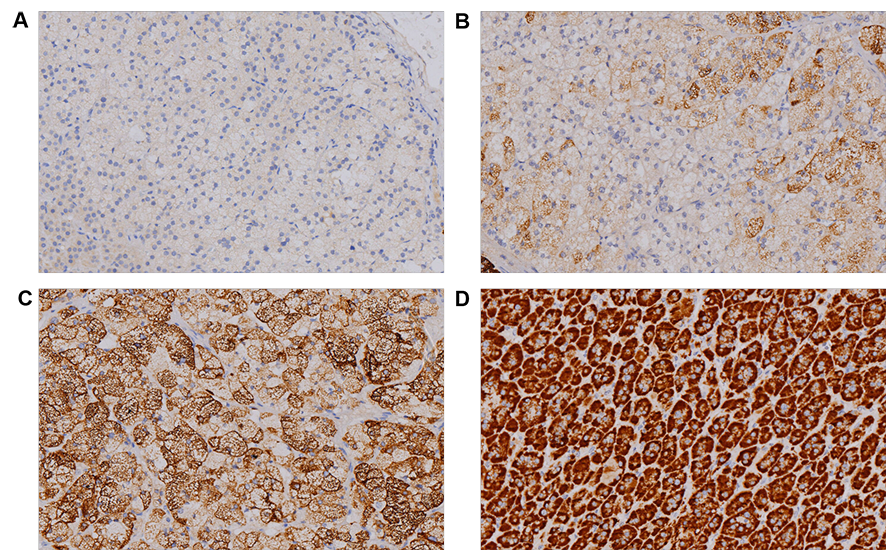

Supplement: Supplementary Figure 3 — The assessment of CPY11B2 expression by immunohistochemistry. (A) Not present (0); (B) weak but detectable above control (1+); (C) distinct (2+); and (D) very strong (3+). [file Image_3.tif]
